# Genomic profiling and comparative analysis of male versus female metastatic breast cancer across subtypes

**DOI:** 10.1186/s13058-024-01872-z

**Published:** 2024-07-24

**Authors:** Arun Kadamkulam Syriac, Nitish Singh Nandu, Allison Clark, Mehrad Tavallai, Dexter X. Jin, Ethan Sokol, Kimberly McGregor, Jeffrey S. Ross, Natalie Danziger, Jose Pablo Leone

**Affiliations:** 1 Dana-Farber Cancer Institute at St. Elizabeth’s Medical Center, Boston, MA USA; 2 University Hospital, University of Missouri Health Care, Columbia, MO USA; 3 Foundation Medicine, Cambridge, MA USA; 4 Upstate Medical University, Syracuse, NY USA; 5 Medical Oncology, Dana-Farber Cancer Institute, 450 Brookline Ave., Boston, MA 02215 USA; 6 Breast Oncology Program, Dana-Farber Brigham Cancer Center, Boston, MA USA; 7 Harvard Medical School, Boston, MA USA; 8 Present Address: Mass General Cancer Center at Wentworth-Douglass Hospital, Dover, NH USA

**Keywords:** Precision medicine, Molecular, Sequencing, Target, Targetable

## Abstract

**Background:**

Male breast cancer (MaBC) has limited data on genomic alterations. We aimed to comprehensively describe and compare MaBC’s genomics with female breast cancer’s (FBC) across subtypes.

**Methods:**

Using genomic data from Foundation Medicine, we categorized 253 MaBC into estrogen receptor (ER)-positive/human epidermal growth factor receptor 2 (HER2)-negative (n = 210), ER-positive/HER2-positive (n = 22) and triple-negative (n = 20). One ER-negative/HER2-positive case was excluded due to n-of-1. The genomics of the final MaBC cohort (n = 252) were compared to a FBC cohort (n = 2708) stratified by molecular subtype, with adjusted *p*-values. In the overall MaBC and FBC cohorts, we compared mutational prevalence in cancer susceptibility genes (CSG) (*ATM/BRCA1/BRCA2/CHEK2/PALB2*).

**Results:**

Comparing ER-positive/HER2-negative cases, MaBc had increased alterations in *GATA3* (26.2% vs. 15.9%, *p* = 0.005), *BRCA2* (13.8% vs. 5.3%, *p* < 0.001), *MDM2* (13.3% vs. 6.14%, *p* = 0.004) and *CDK4* (7.1% vs. 1.8%, *p* < 0.001); and decreased frequency of *TP53* (11.0% vs. 42.6%, *p* < 0.001) and *ESR1* mutations (5.7% vs. 14.6%, *p* < 0.001). Comparing ER-positive/HER2-positive cases, MaBC had increased short variants in *ERBB2* (22.7% vs. 0.6%, *p* = 0.002), *GATA3* (36.3% vs. 6.2%, *p* = 0.004), and *MDM2* (36.3% vs. 4.9%, *p* = 0.002); decreased frequency of *TP53* alterations was seen in MaBC versus FBC (9.1% vs. 61.7%, *p* < 0.001). Within triple-negative cases, MaBC had decreased alterations in *TP53* compared to FBC (25.0% vs. 84.4%, *p* < 0.001). MaBC had higher frequency of CSG variants than FBC (22.6% vs. 14.6%, *p* < 0.05), with increased *BRCA* mutations in MaBC (14.6% vs. 9.1%, *p* < 0.05).

**Conclusions:**

Although MaBC and FBC share some common alterations, our study revealed several important differences relevant to tumor biology and implications for targeted therapies.

**Supplementary Information:**

The online version contains supplementary material available at 10.1186/s13058-024-01872-z.

## Introduction

Male breast cancer (MaBC) is a rare entity. It is estimated that about 2,800 new cases of MaBC will be diagnosed in 2023, and about 530 men will die from breast cancer [1]. Compared with female breast cancer (FBC), the incidence rate per 100,000 is significantly lower (1.28 vs. 125.11) [2]. Due to the absence of routine mammographic screening, MaBC is often diagnosed with a more advanced stage than FBC [3, 4]. However, when adjusting for age of diagnosis, tumor subtype, and stage, overall survival rates for MaBC are comparable with FBC [5].

The genomic landscape of MaBC has not been fully characterized. In a large study involving 1483 MaBC samples, most were estrogen receptor (ER)-positive (99%), progesterone receptor-positive (82%), and/or androgen receptor-positive (97%). The majority had a luminal B-like/human epidermal growth factor receptor 2 (HER2)-negative (48.6%) or a luminal A-like (41.9%) phenotype. Only 9% of tumors were HER2-positive, and < 1% were triple-negative breast cancer (TNBC) [6]. Piscuoglio et al. examined 59 MaBC cases and reported 29% of those as luminal A-like and 71% as luminal B-like. In addition, they reported similar types of mutations between MaBC and FBC, but the frequencies were different. *PIK3CA* mutations, *TP53*, and 16q losses were less frequent in MaBC [7]. Moelans et al. studied somatic mutations of 135 cases of MaBC and demonstrated that *PIK3CA, KMT2C, PBRM1*, and *GATA3* were the most frequently mutated genes [8].

Due to the rarity of MaBC, limited data exist on genomic alterations and the prevalence of breast cancer susceptibility genes (CSG). We aimed to comprehensively describe the genomics of MaBC and compare these to a FBC cohort across molecular subtypes to provide insight into tumor biology and opportunities for targeted therapies.

## Materials and methods

Approval for this study, including a waiver of informed consent and a HIPAA waiver of authorization, was obtained from the Western Institutional Review Board (Protocol No. 20152817). The study was conducted in accordance with the Declaration of Helsinki. A total of 337 MaBC tissue biopsies from patients with diagnosis of metastatic breast cancer were sequenced by Foundation Medicine using hybrid capture-based comprehensive genomic profiling (CGP).

The assay was performed on patient samples in a Clinical Laboratory Improvement Amendments (CLIA)–certified, College of American Pathologists (CAP)-accredited, New York State–approved laboratory (Foundation Medicine, Cambridge, MA).

Tissue-based testing evaluated 324–395 cancer-related genes. The testing assessed base substitutions, short insertions/deletions, rearrangements/fusions, and copy number alterations (amplifications and deep deletions) [9].

**Fig. 1:**
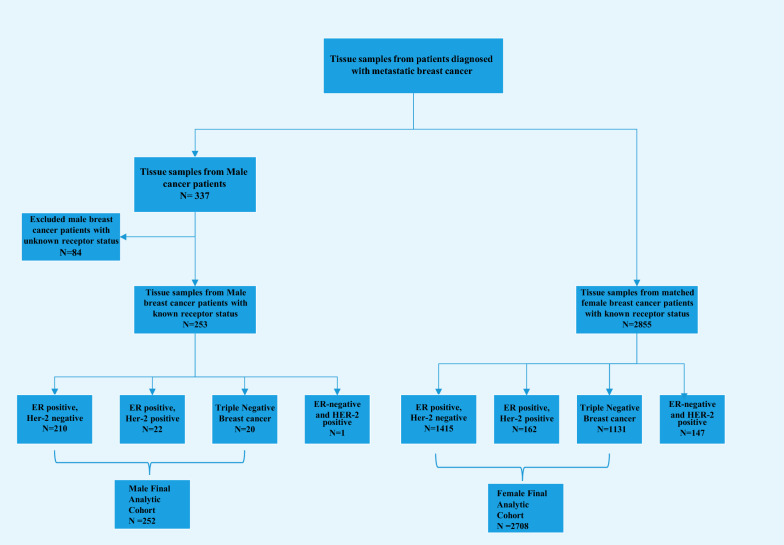
Flow diagram for the study population. ER, estrogen receptor; HER2, human epidermal growth factor receptor 2

Detection of CSGs was conducted using a previously published approach [10]. Mutational prevalence in 5 breast CSG (*ATM/BRCA1/BRCA2/CHEK2/PALB2*) was compared, along with their associated genomic loss of heterozygosity (gLOH) values [11, 12]. Diagnosis, biopsy site, and the date of specimen collection were extracted from test requisition forms and pathology reports. Given the rarity of MaBC, and in particular certain molecular subtypes of this disease, the study pathologist (JSR) did a pathology review of the male cases for diagnostic accuracy. Cases were classified based on ER status and HER2 amplification data. The MaBC cohort with known ER and HER2 amplification status (n = 253) was compared to a FBC cohort (n = 2855) from the Foundation Medicine database. Inclusion criteria were the same for both MaBC and FBC cohorts and consisted of cases submitted to Foundation Medicine for sequencing that achieved successful sequencing results, on whom we had available information regarding ER and HER2 status. The classification of HER2-low cases in MaBC was done via manual review by the study pathologist (JSR). The sample distribution and cohort selection are shown in Fig. 1.

### Statistical analysis

Univariate comparisons of proportion were made using a Fisher’s exact test. False discovery rate adjusted *p*-values were calculated using the Benjamini–Hochberg procedure, and all *p*-values presented are adjusted *p*-values. Results were considered statistically significant when adjusted *p*-values were < 0.05. Genomic profiling results were compared between men and women within 3 specific molecular subtypes: ER-positive/HER2-negative, ER-positive/HER2-positive, and ER-negative/HER2-negative (TNBC) subtypes. Given that we had only one MaBC sample that was ER-negative and HER2-positive, this subtype was excluded from comparison with women and excluded from further analysis (Fig. 1). For the comparison of gLOH, continuous distributions were compared using a non-parametric Mann–Whitney U test.

## Results

**Fig. 2:**
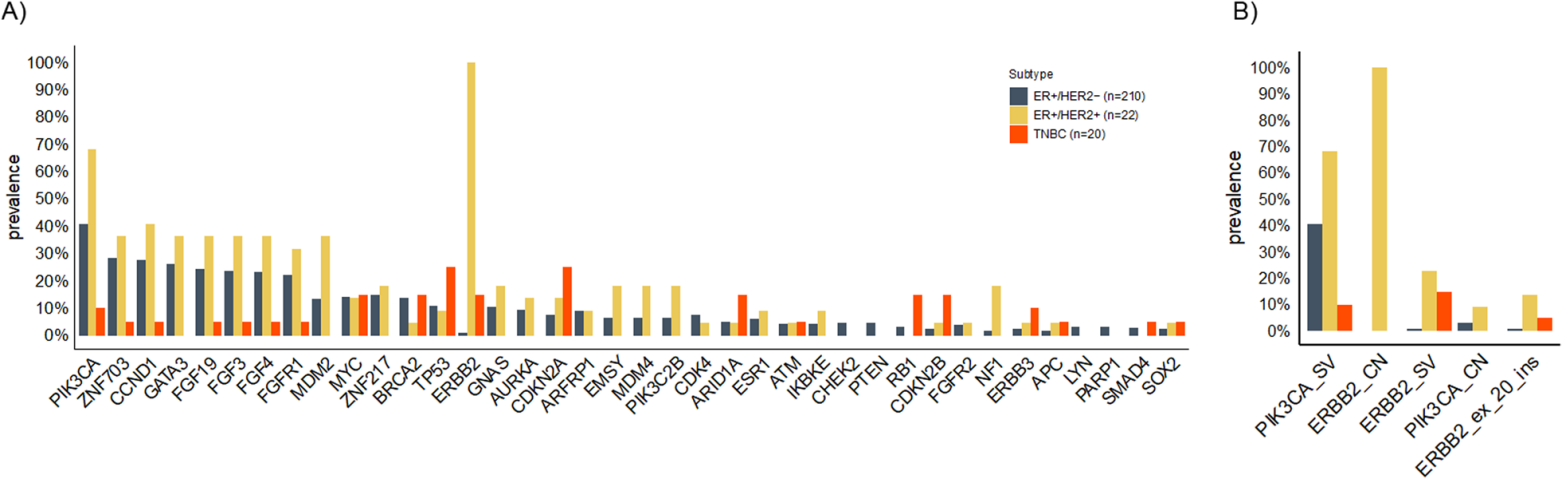
Landscape of genomic alterations in the male breast cancer (MaBC) cohort by molecular subtype. The X axis lists the individual genes depicted with their alteration prevalence on the Y axis. **a** Distribution of genomic alterations in the MaBC cohort. **b** Distribution of genomic alterations of special interest. CN, copy number; ER, estrogen receptor; ex_20_ins, exon 20 insertion; HER2, human epidermal growth factor receptor 2; SV, somatic variant; TNBC, triple-negative breast cancer

A total of 337 MaBC samples were analyzed for this study. Of these, 253 tissue biopsy samples had known ER and HER2 status. ER-positive/HER2-negative was the most common subtype and was observed in 83% of samples (n = 210), 8.7% of samples (n = 22) were ER-positive/HER2-positive, 7.9% (n = 20) were TNBC, and 0.4% (n = 1) were ER-negative/HER2-positive. These samples were compared to a female cohort (n = 2855) with known ER and HER2 status. The distribution of molecular subtypes in the FBC cohort was: 49.6% ER-positive/HER2-negative (n = 1415), 5.7% ER-positive/HER2-positive (n = 162), 39.6% TNBC (n = 1131), and 5.1% ER-negative/HER2-positive (n = 147) (Fig. 1). We excluded the ER-negative/HER2-positive subgroup from both cohorts in our genomic analyses. Additional file 1: Figure S1 shows the source of sample collection by molecular subtype for both the male and female cohorts. The landscape of genomic alterations in the MaBC cohort by molecular subtype is shown in Fig. 2.

### Comparison of MaBC and FBC genomics

Using the final analytic cohorts (MaBC, n = 252; FBC, n = 2708), we analyzed the distinctive genomic features of MaBC relative to FBC by tumor subtype. Table 1 summarizes the comparison of gene alterations between the cohorts by tumor subtype.

**Table 1:** Comparison of relevant gene alterations between male and female breast cancer by molecular subtype

List of genes by molecular subtype	Male	Female	*p*
*ER-positive/HER2-negative subgroup*	(*n* = 210)	(*n* = 1415)	
GATA3	26.2%	15.9%	Adjusted *p* = 0.005
TP53	10.9%	42.6%	Adjusted *p* < 0.001
MDM2	13.3%	6.1%	Adjusted *p* = 0.004
CDK4	7.1%	1.8%	Adjusted* p* < 0.001
BRCA2	13.8%	5.4%	Adjusted* p* < 0.001
ESR1	5.7%	14.6%	Adjusted *p* < 0.001
*ER-positive/HER2-positive subgroup*	(*n* = 22)	(*n* = 1131)	
ERBB2 short variants	22.7%	0.6%	Adjusted *p* = 0.002
PIK3CA	68.2%	37.0%	Adjusted *p* = 0.1
GATA3	36.3%	6.2%	Adjusted *p* = 0.004
MDM2	36.3%	4.9%	Adjusted* p* = 0.002
TP53	9.1%	61.7%	Adjusted *p* < 0.001
*Triple-negative breast cancer subgroup*	(*n* = 20)	(*n* = 162)	
TP53	25%	84.4%	Adjusted* p* < 0.001
BRCA2	15%	4.0%	Adjusted *p* = 0.3

ER, estrogen receptor; HER2, human epidermal growth factor receptor 2

#### ER-positive/HER2-negative subgroups

**Fig. 3:**
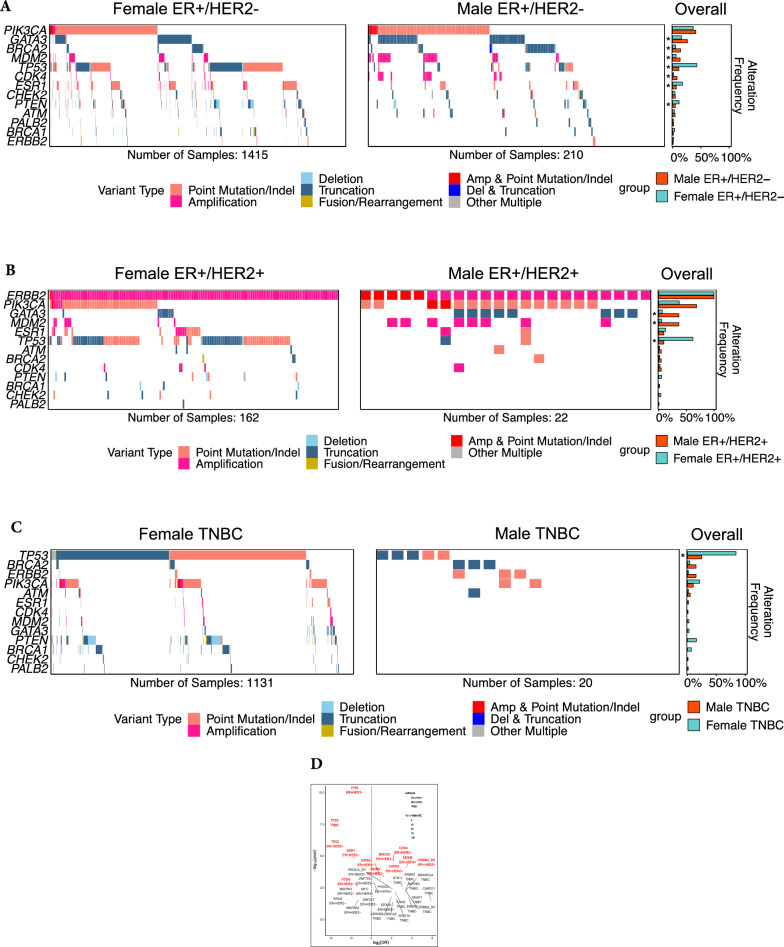
Comparison of genomic alterations between male and female breast cancer cohorts by subtype and genes with differences in alteration frequency by cohort. **a** Comparison of genomic alterations between male (on the right) and female (on the left) breast cancer cohort of estrogen receptor (ER)-positive/human epidermal growth factor receptor (HER2)-negative subtypes. Asterisks reflect adjusted *p* < 0.05. Frequencies of alterations for MaBC and FBC include: *GATA3*: 26.2% vs. 15.9%; *BRCA2*: 13.8% vs. 5.4%; *MDM2*: 13.3% vs. 6.1%; *TP53*: 11.0% vs. 42.7%; respectively. **b** Comparison of genomic alterations between male (on the right) and female (on the left) breast cancer cohorts of ER-positive/HER2-positive subtypes. Asterisks reflect adjusted *p* < 0.05. Frequencies of alterations for MaBC and FBC include: *GATA3*: 36.4% versus 6.2%; *MDM2*: 36.4% versus 4.9%; *TP53*: 9.1% versus 61.7%; respectively. **c** Comparison of genomic alterations between male (on the right) and female (on the left) breast cancer cohorts of Triple-Negative Breast Cancer (TNBC) subtypes. Asterisk reflects adjusted *p* < 0.05. Frequencies of alterations for MaBC and FBC include: *TP53*: 25.0% versus 84.4%; respectively. **d** Genes with differences in alteration frequency in male and female breast cancer. Y-axis depicts the significance (negative of the log10 transformed *p*-value) and X-axis depicts the log2 transformed odds ratio. The dotted line on the x-axis demarcates depletion versus enrichment, where values < 0 indicate depletion and values > 0 indicate enrichment. Red text = False Discovery Rate (FDR) < 0.05, black text = *p* < 0.05, but FDR >  = 0.05. ER, estrogen receptor; HER2, human epidermal growth factor receptor 2

In ER-positive/HER2-negative cohorts, we observed an increased frequency of alterations in *GATA3* (26.2% in males vs. 15.9% in females, adjusted *p* = 0.005), *MDM2* amplifications (13.3% vs. 6.14%; adjusted *p* = 0.004), *CDK4* amplifications (7.1% vs. 1.8%; adjusted *p* < 0.001), and *BRCA2* alterations (13.8% vs. 5.3%; adjusted *p* < 0.001) in men relative to women (Fig. 3a, d).

The ER-positive/HER2-negative MaBC group displayed a decreased frequency of *TP53* and *ESR1* alterations compared with women of the same subtype [11.0% vs. 42.6% for *TP53*, adjusted *p* < 0.001; and 14.6% vs. 5.7% for *ESR1*, adjusted *p* < 0.001 (Fig. 3a, d)].

#### ER-positive/HER2-positive subgroups

Within the ER positive/HER2-positive subgroups, an increase in *ERBB2* short variants was observed in the male cohort (22.7%, n = 5) compared to the female cohort (0.6%, adjusted *p* = 0.002) (Fig. 3b, d). Of the 5 male samples with *ERBB2* short variants, three were *ERBB2* exon 20 insertions and two were L755S. All five of these are kinase domain impacting. We observed significant differences in *ERBB2* copy number distribution between male and females. MaBC had a median *ERBB2* copy number of 9 (IQR: 7–20.75) as compared with the median *ERBB2* copy number of 19 (IQR: 8–44) in FBC (*p* = 0.006) (Additional file 1: Figure S2).

There was a significantly higher frequency of alterations in the *GATA3* gene (36.3% male vs. 6.2% female; adjusted *p* = 0.004), and *MDM2* alterations (36.3% male vs. 4.9% female; adjusted *p* = 0.002) when compared to the cohort of FBC patients (Fig. 3b, d). In addition, we observed a nominally higher frequency of *PIK3CA* alterations in male patients (68.18%) compared to the female patients (36.46%) (unadjusted P = 0.009 but adjusted P = 0.1) (Fig. 3b, d).

We observed a higher frequency of alterations of *TP53* in the female cohort (61.7%) compared to the male cohort (9.1%) with an adjusted *p* < 0.001; (Fig. 3b, d).

#### TNBC subgroups

In the TNBC subgroup, there was a nominally increased frequency of *BRCA2* alterations in the male cohort compared to the female cohort, however, this finding was not statistically significant after multiple hypothesis correction [15.0% vs. 4.0% respectively unadjusted *p* = 0.047 and adjusted *p* = 0.3 (Fig. 3c, d)]. A significantly higher frequency of *TP53* alterations were seen in the female TNBC cohort as compared to the male cohort [84.4% vs. 25.0% respectively; adjusted *p* < 0.001 (Fig. 3c, d)].

#### HER2-low in MaBC

As a descriptive exploratory analysis, we aimed to assess the frequency of HER2-low cases in our study population in light of the recent data from trastuzumab deruxtecan [13]. We found that among HER2-negative cases, a total of 66.9% would have met eligibility criteria for Destiny Breast 04 (as defined by immunohistochemistry [IHC] 1+ or IHC 2+ / in situ hybridization negative).

### Breast *cancer* susceptibility genes (CSG) in MaBC

Among the final analytic cohorts (MaBC, n = 252; FBC, n = 2708), there was a higher prevalence of alterations in at least one of the 5 breast cancer associated CSGs of potential germline origin seen in males compared to females (22.6% vs. 14.6% respectively, *p* = 0.0014). There was also a higher percentage of *BRCA* 1 and 2 mutations in male patients compared to female patients with breast cancer (14.6% vs. 9.1%, *p* = 0.0006).

**Fig. 4:**
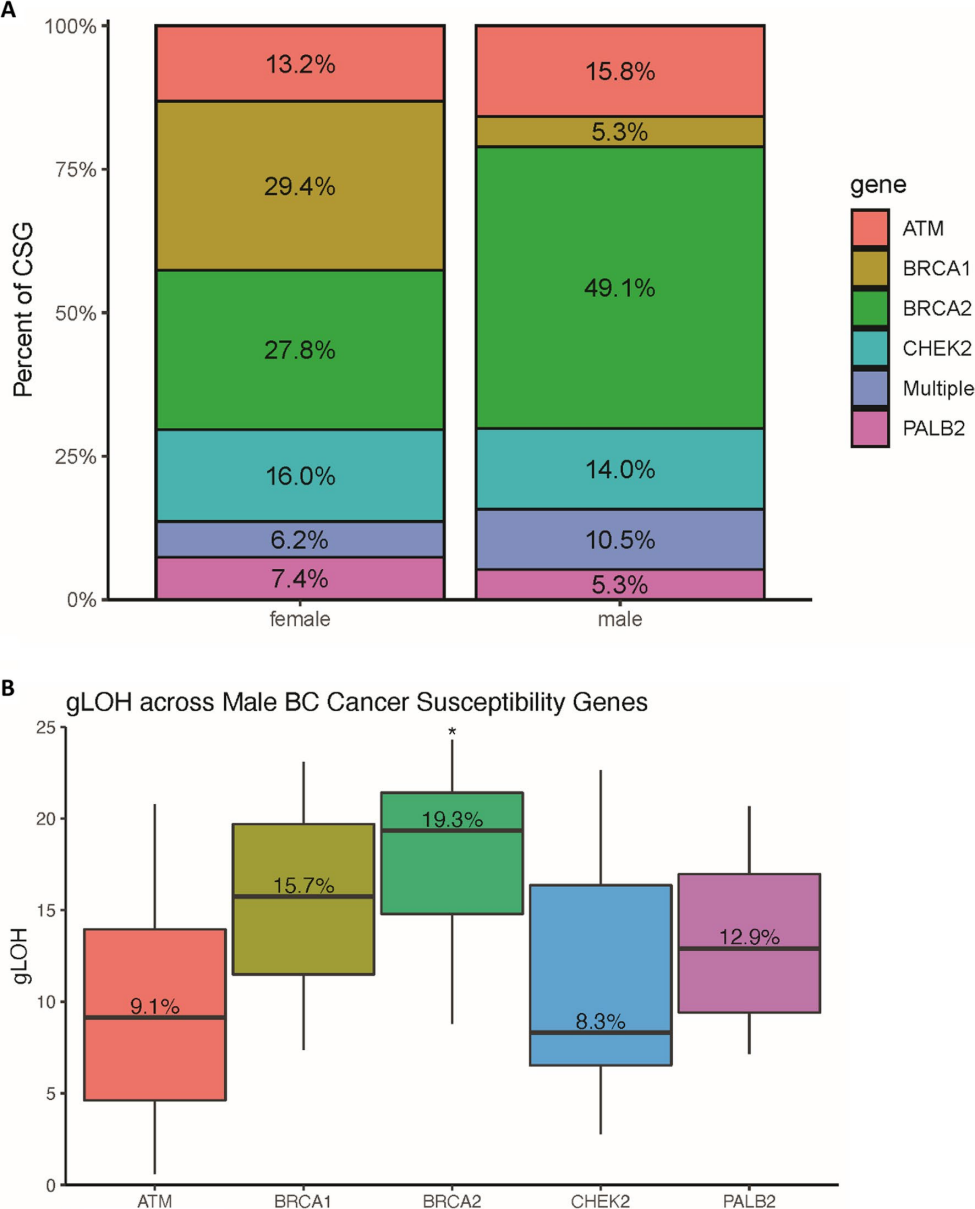
Comparison of breast cancer susceptibility genes (CSG) in male versus female breast cancer and genome-wide loss of heterozygosity (gLOH) in male breast cancer. **a** Fraction of breast cancer susceptibility genes (CSG) in male (n = 57) and female (n = 418) breast cancer patients. **b** Median genome-wide loss of heterozygosity (gLOH) across male breast cancer (MaBC) patients, stratified by breast CSG gene prevalence. Asterisk reflects adjusted *p* < 0.05

Among the 57 men with an alteration in at least one of the five CSG, *BRCA2* mutations were the most prevalent alteration, at 57.9% (n = 33), compared to *BRCA1* which represented 7.0% of alterations (n = 4). In contrast, FBC patients with an alteration in at least one of the five CSG (n = 418) had a similar prevalence of *BRCA2* (31.3%, n = 131) and *BRCA1* (31.6%, n = 132). The prevalence of *CHEK2*, *ATM*, *PALB2* and multiple genes, was proportionally similar in both MaBC and FBC patients. Figure 4a shows the distribution of alterations for the five CSG, including the distribution of cases with more than one CSG, among men and women known to have CSG alterations.

Among MaBC patients, the *BRCA2* gene was associated with the highest median gLOH scores (19.3%), followed by *BRCA1* (15.7%), *PALB2* (12.9%), *ATM* (9.1%), and *CHEK2* (8.3%) genes (Fig. 4b). We compared the gLOH scores for each CSG in samples with CSG alterations versus wild type and found that only *BRCA2* was statistically significant with an adjusted *p* < 0.05 (Fig. 4b denoted with asterisk).

## Discussion

In this study, we evaluated a series of 252 MaBC samples that underwent next-generation sequencing using Foundation Medicine and compared the genomic profiling with a FBC cohort of 2708 cases from the same source, stratified by molecular subtype. While there were genomic similarities between male and female breast cancers, our study showed important genomic differences between the two sexes.

Men with ER-positive/HER2-negative breast cancer had more frequent alterations in *GATA3*, *MDM2*, *CDK4*, *BRCA2* genes as compared with FBC patients of the same subtype. *GATA3* is a defining marker for luminal cancers and an important regulator of luminal differentiation [14, 15]. Mutations in *GATA3* have been associated with changes in luminal biology endocrine resistance, and worse prognosis [16].

The presence of alterations in *BRCA2* in MaBC provides opportunity for targeted therapy with PARP inhibitors. As compared with women, men in our study with ER-positive/HER2-negative breast cancer were also found to have fewer alterations in *TP53* and *ESR1* genes. While intriguing, the lower frequency of *TP53* in men compared with women and the higher frequency of *MDM2* in men compared with women may represent true biologic differences between male and female breast cancer tumor biology. In fact, alterations in *TP53* and *MDM2* are nearly mutually exclusive events and suggest different biological pathways to p53 inactivation. The lower incidence of *ESR1* mutations in men is suggestive of lower rates in use of aromatase inhibitors as compared with women.

When comparing patients with ER-positive/HER2-positive breast cancer, male patients had higher alterations in *ERBB2*, *PIK3CA*, *GATA3*, and *MDM2* genes while female patients had higher alterations in *TP53* genes. Mutations in *ERBB2* and *PIK3CA* are associated with resistance to anti-HER2 therapies [17–21].

Two prior studies have shown that, as compared with women, men with HER2-positive breast cancer have worse survival [5, 22]. A recent analysis of National Cancer Database showed that men with HER2-positive breast cancer have 60% lower odds of achieving pathologic complete response to neoadjuvant chemotherapy compared with women of the same subtype [23, 24]. Taken together, the data from the aforementioned manuscripts show that HER2-positive breast cancer in men appears to have worse prognosis than in women. Our current study suggests that the differences in prognosis may be related to genomic alterations that confer resistance to anti-HER2 therapy, such as *ERBB2* short variants and *PIK3CA* alterations, both of which are associated with therapy resistance and poor prognosis. Interestingly, our study showed that the median *ERBB2* copy number in MaBC is significantly lower than in FBC, highlighting additional molecular differences in HER2-positive breast cancer between males and females which may impact sensitivity to anti-HER2 therapy. It is possible that HER2-positive MaBC may be less HER2 addicted than HER2-positive FBC.

Among patients with TNBC, a higher frequency of alterations in the *BRCA2* gene was observed in male patients. We also observed a significantly higher frequency of alterations in *TP53* in female patients when compared to male patients. *BRCA2* has a critical role in DNA repair, and mutations in *BRCA2* are a known risk factor for the development of breast cancer in men.^3^ Two pivotal trials demonstrated the role of PARP inhibitors in the treatment of *BRCA-*mutated metastatic breast cancer [25, 26]. Our results suggest that alterations in *BRCA2* are very prevalent in MaBC and raise the possibility of using PARP inhibitors in this population.

As observed in our study, there are important genomic differences between MaBC and FBC. One possible explanation for the different genomic alteration frequencies may be differences in intrinsic subtypes between sexes. In fact, analysis of genomic intrinsic subtypes in male breast cancer has shown a predominant luminal disease, with higher frequency of luminal B tumors and lower frequency of HER2 enriched and basal-like tumors [27].

Our study revealed a considerable number of men with breast CSG alterations (22.6%), which was significantly higher than in women (14.6%). Similarly, *BRCA* mutations were more frequent in men than in women. This represents a significant opportunity for targeted therapy with PARP inhibitors. The phase III EMBRACA trial (25) and Olympiad trial (26) showed improvements in progression-free survival in *BRCA 1/2-*mutated HER2-negative population. Notably, there were only 5 men in the treatment arm of the Olympiad trial, and 2 in the trial’s standard therapy group. Similarly, men in the EMBRACA trial represented less than 2% of the study population. As expected, we observed that *BRCA2* was the predominant alteration in men, consistent with prior studies [28–31]. To our knowledge, our study represents the largest analysis of breast cancer susceptibility genes in MaBC to date.

Our study had some important limitations. The first is that the study is retrospective. Secondly, we do not have patients’ clinical information including some demographics, treatment, and clinical outcomes. Some alteration frequencies reported may be impacted by the sample used for sequencing, as the mutational spectrum can change over time; in this regard, while the tissue sources are described in Additional file 1: Figure S1, the lines of therapy that patients received before sample acquisition are unknown. This is an important limitation considering that prior treatment may alter alteration frequencies. Given the lack of information on some patient characteristics and prior treatments, there may be unmeasured differences between the male and female cohorts. The sample size of HER2-positive and triple-negative MaBC was small, and we had to exclude the one case that was ER-negative/HER2-positive. This underrepresentation is expected owing to the rarity of these subtypes in men [32]; nonetheless, caution should be taken when interpreting the results from these smaller subgroups. Unfortunately, data on HER2-low classification was not available for the FBC cohort and thus prevents comparisons with the findings of the MaBC cohort. Additionally, our study lacked matched normal tissue to help determine whether aberrations are germline or somatic, and lacked information on variant allele frequencies. Finally, given that we are reporting on cases that were specifically submitted for genomic analysis, our study has a selection bias and the study population may not be representative of the overall population of men with breast cancer. To address this issue, we used a female cohort with the same inclusion criteria and from the same source, likely sharing the same degree of selection bias in the control group.

Despite these limitations, there are several important strengths to our study. To our knowledge, this is the largest reported cohort of MaBC undergoing genomic analysis. In addition, we have included a comprehensive approach consisting of extensive genomic characterization with a high number of genes, including breast CSGs, which if confirmed as germline can have additional impact on these patients and their families. We compared genomic alterations between male and female by molecular subtype, which provides subtype-specific information that is clinically more relevant. Our study results provide important information about clinically actionable alterations in MaBC with regards to somatic mutations, as well as breast CSGs.

## Conclusions

We observed that, when compared with FBC, MaBC has an increased frequency of alterations in *GATA3* and *MDM2* and fewer alterations in *TP53*. We also noticed increased rates of *ERBB2* short variants, *PIK3CA* alterations, *BRCA2* mutations, and other breast CSG alterations that were more common in MaBC. The landscape of MaBC can help identify targeted therapies and better understand tumor biology.

### Supplementary Information

Additional file 1

## Data Availability

The sequencing data generated in this study were derived from clinical samples. The data supporting the findings of this study are provided within the paper and its supplementary files. Due to HIPAA requirements, we are not consented to share individualized patient genomic data, which contains potentially identifying or sensitive patient information. Foundation Medicine is committed to collaborative data analysis, and we have well-established, and widely utilized mechanisms by which investigators can query our core genomic database of > 600,000 de-identified sequenced cancers to obtain aggregated datasets. Requests for collaborative datashares can be made by contacting the corresponding author(s) and filling out a study review committee form. Once approved, investigators are required to sign a data transfer agreement. Written proposals are considered at monthly meetings and data transfer agreements expire 18 months from execution of the agreement.

## Abbreviations

MaBC: Male breast cancer
FBC: Female breast cancer
ER: Estrogen receptor
HER2: Human epidermal growth factor receptor 2
TNBC: Triple-negative breast cancer
CSG: Cancer susceptibility genes
CGP: Comprehensive genomic profiling
CLIA: Clinical Laboratory Improvement Amendments
CAP: College of American Pathologists
gLOH: Genomic loss of heterozygosity
IHC: Immunohistochemistry
