# Supplementary material for: Genomic profiling and comparative analysis of male versus female metastatic breast cancer across subtypes
Source: Breast Cancer Res. 2024 Jul 24;26:118. doi: 10.1186/s13058-024-01872-z (PMC11267671; doi:10.1186/s13058-024-01872-z)

Additional File:
“Genomic profiling and comparative analysis of male vs. female metastatic breast cancer across subtypes"

Arun Kadamkulam Syriac, Nitish Singh Nandu Allison Clark, Mehrad Tavallai, Dexter X. Jin, Ethan Sokol, Kimberly McGregor, Jeffrey S. Ross, Natalie Danziger, and Jose Pablo Leone

This file contains the following items:

- **Supplementary Figure S1:** Sample site by molecular subtype shown for both the male and female cohorts.
- **Supplementary Figure S2:** Distribution of ERBB2 copy number between males and females with breast cancer. The figure depicts median copy number values and interquartile range

Supplementary Figure S1: Sample site by molecular subtype shown for both the male and female cohorts.

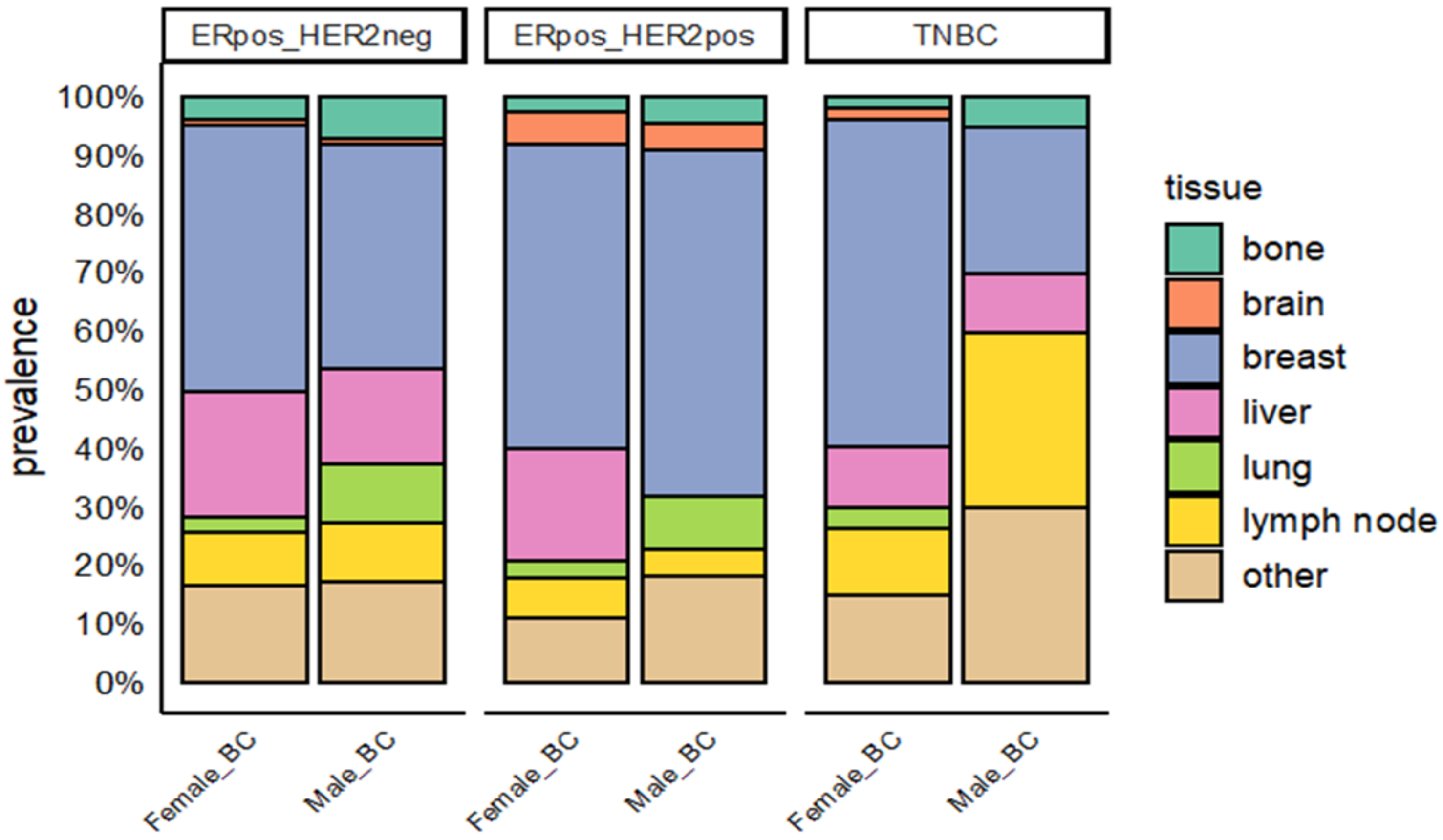

Supplementary Figure S2: Distribution of ERBB2 copy number between males and females with breast cancer. The figure depicts median copy number values and interquartile range.

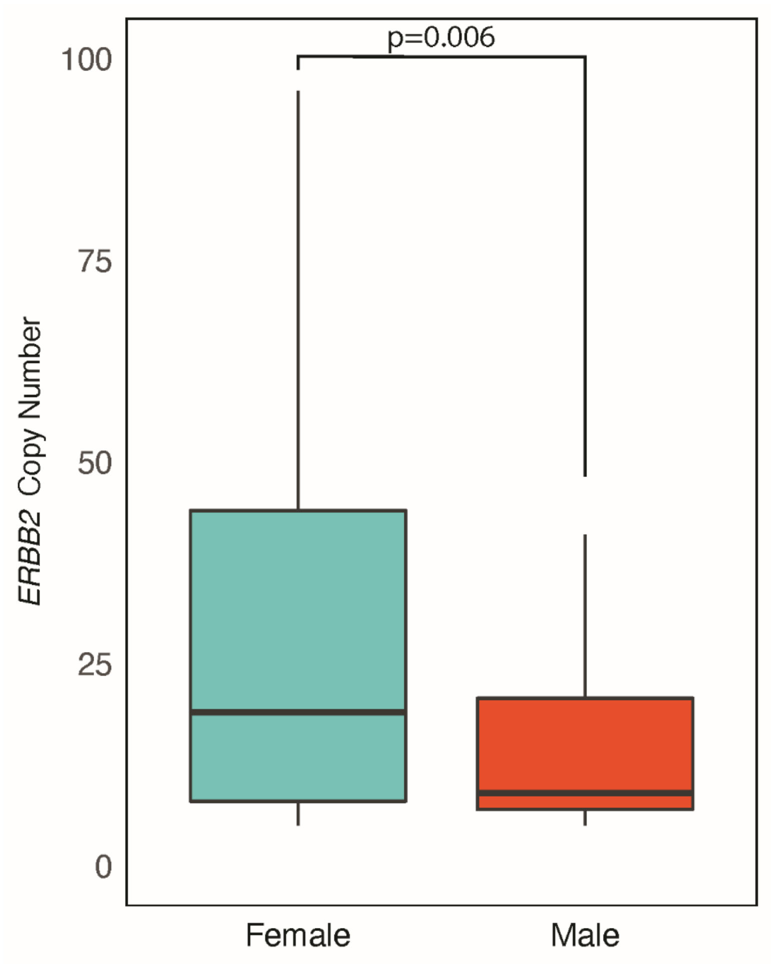

Supplement: Supplementary file 1 — Additional file 1 [file 13058_2024_1872_MOESM1_ESM.pdf]
